# Torsion of non-gravid uterus with ovarian cyst - an extremely rare case

**DOI:** 10.11604/pamj.2014.18.95.1430

**Published:** 2014-05-27

**Authors:** Nusrat Havaldar, Kiran Ashok

**Affiliations:** 1 Department of Obstetrics and Gynecology, PESIMSR, Kuppam, Andhra Pradesh, India; 2 ESI Hospital, Bangalore, India

**Keywords:** Torsion, nongravid uterus, ovarian cyst

## Abstract

Torsion of nongravid uterus is rare. Cases reported have occured during pregnancy. We report a case of a patient with acute abdominal pain. The patient showed a voluminous mass situated in the abdomen and noncontiguous to the uterus by ultrasound scan. Laparotomy confirmed the diagnosis of cystic ovarian mass with torsion of uterus.

## Introduction

Uterine torsion is a rare condition in the nongravid uterus [1]. Uterine torsion may cause irreversible ischaemic damage to the uterus, leading to rapid clinical deterioration, [2]. In non-pregnant women, this condition has only been reported in only a few cases, the first by The Times in 1861, followed by other cases in the twentieth century. Diagnosis is frequently made intraoperatively, mainly due to poor correlation between clinical symptoms and classic radiological findings. The leading causes are the presence of leiomyomas or, rarely, adnexal masses. In this report a 55-year old patient presented with acute abdominal pain. After the gynecologic examination and imaging evaluation, it was found to be a pelvic mass suggesting hemorrhagic ovarian.

## Patient and observation

**Figure 1:**
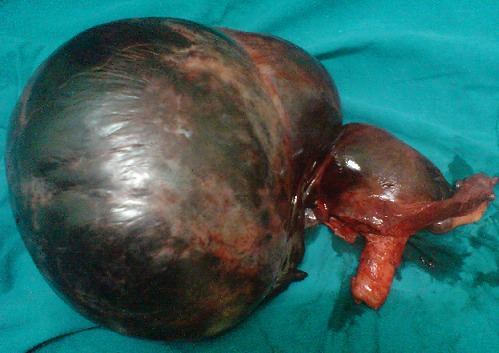
Gross specimen

A 55 year old multiparous postmenopausal lady presented to casualty with severe pain abdomen for the past 2 days which was sudden in onset,continuous,associated with 2 episodes of vomiting,not passed stools and flatus in the last 2 days. There was no history of fever, bleeding per vagina or per rectum, previous surgeries, discharge per vagina or bladder disturbances. Patient was conscious, oriented and in severe pain. On abdominal examination there was distension with a tense, tender, cystic swelling arising from pelvis more on the right side, about 20x20 cm size. Cervix not seen on per speculum examination. On per vaginum uterus not separately made out,cervix high up behind symphysis pubis. On per rectal examination pouch of douglas was free,rectal mucosa free. On investigating ultrasonography showed a large cystic mass of 21x19cms with septae and internal echoes suggestive of ovarian cyst with hemorrhage. Examination under anesthesia revealed two separate masses, one in the right iliac fossa & the other in the suprapubic region with a groove in between them. A laparotomy was performed by vertical midline incision, and we found a 20x20cms right cystic ovarian mass with torsion of three turns involving infundibulopelvic ligament and ovarian ligament. Uterus had undergone 1800 rotation at the level of isthmus (round ligaments were in antero-posterior direction rather than normal transverse). The left tube and ovary were normal. A total abdominal hysterectomy with bilateral salpingo oopherectomy was performed (Figure 1). The upper two thirds of the uterus was congested and bluish in color incontrast to healthy cervix which indicates torsion at the level of the isthmus.

## Discussion

Uterine torsion is defined as rotation of the uterus on its long axis by more than 45 degrees [3]. The position of the uterus is stabilised by the broad ligaments and the uterosacral ligaments [4]. It is difficult to explain the mechanism of an axial rotation for normal uterus,though it appears that uterine axial torsion is usually caused by the presence of pathological or abnormal condition in the uterus or the adjacent structures, uterine fibroids being the most common predisposing factor. Two cases of nongravid non myomatous uterine torsion have also been described. Uterine torsion is a rare condition. Although it has been reported since 1861 [4]. only about 200 cases have been reported in the past 100 years. Most reported cases involved gravid uteri, thus torsion of the nongravid uterus is even more rare. For this reason, the clinical course, prognosis, and mortality rates associated with torsion of a nongravid uterus are not well documented. Uterine torsion is difficult to diagnose preoperatively [5]. This may be due to lack of symptoms specific to the condition. The clinical presentation varies from non-specific mild abdominal discomfort to acute abdomen with shock [6]. Our patient presented with non-specific symptoms (abdominal pain, distension, vomiting) rendering a prompt and accurate diagnosis of uterine torsion extremely difficult. Specific clinical signs including vaginal bleeding, uterine tenderness, a twisted vaginal canal and urethral displacement have been reported. The differential diagnosis should include appendicitis, fibroid degeneration, adnexal torsion, abdominal pregnancy, and placental abruption. Management guidelines of an acute symptomatic patient is laparotomy following initial assessment, possibly after a short period of observation. Hysterectomy should be considered in cases of prolonged torsion with subsequent necrosis and blood thrombosis. For uncomplicated and carefully selected cases with no necrosis a conservative surgical approach was possible [7]. In our case there was necrosis and also the patient was postmenopausal hence a hysterectomy with bilateral salpingo oopherectomy was performed.

## Conclusion

Torsion of the nongravid uterus is an uncommon condition. Delay in diagnosing this condition may prove fatal, hence there is a need for a high index of suspicion. Uterine torsion should be considered as a differential diagnosis in women presenting with acute abdominal pain.

## References

[1] Omurtag K, Session D, Brahma P, Matlack A, Roberts C (2009). Horizontal uterine torsion in the setting of complete cervical and partial vaginal agenesis: a case report. Fertil Steril.

[2] Grover S, Sharma Y, Mittal S (2009). Uterine torsion: a missed diagnosis in young girls?. J Pediatr Adolesc Gynecol..

[3] Jeong YY, Kang HK, Park JG, Choi HS (2003). CT features of uterine torsion. Eur Radiol..

[4] Hawes CH (1935). Acute axial torsion of the uterus. Ann Surg..

[5] Nicholson W, Coulson CC, McCoy MC, Semelka RC (1995). Pelvic magnetic resonance imaging in the evaluation of uterine torsion. Obstet Gynecol..

[6] Dua A, Fishwick K, Deverashetty B (2006). Uterine torsion in pregnancy: a review. The Internet Journal of Gynecology and Obstetrics..

[7] Collinet P, Narducci F, Stien L (2001). Torsion of a nongravid uterus: an unexpected complication of an ovarian cyst. Eur J Obstet Gynecol Reprod Biol..

